# Mapping Epitopes Recognised by Autoantibodies Shows Potential for the Diagnosis of High-Grade Serous Ovarian Cancer and Monitoring Response to Therapy for This Malignancy

**DOI:** 10.3390/cancers13164201

**Published:** 2021-08-20

**Authors:** Rhiane Moody, Kirsty Wilson, Nirmala Chandralega Kampan, Orla M. McNally, Thomas W. Jobling, Anthony Jaworowski, Andrew N. Stephens, Magdalena Plebanski

**Affiliations:** 1 School of Health and Biomedical Sciences, RMIT University, Bundoora, VIC 3083, Australia; s3740510@student.rmit.edu.au (R.M.); kirsty.wilson2@rmit.edu.au (K.W.); anthony.jaworowski@rmit.edu.au (A.J.); 2 Department of Obstetrics and Gynaecology, Universiti Kebangsaan Malaysia Medical Centre, Kuala Lumpur 56000, Malaysia; nirmala@ukm.edu.my; 3 Gynaeoncology Unit, Royal Women’s Hospital, Parkville, VIC 3052, Australia; Orla.McNally@thewomens.org.au; 4 Department of Gynaecological Oncology, Monash Medical Centre, Bentleigh East, VIC 3165, Australia; tjobling@bigpond.net.au; 5 Hudson Institute of Medical Research, Clayton, VIC 3168, Australia; andrew.n.stephens@hudson.org.au; 6 Department of Molecular and Translational Sciences, Monash University, Clayton, VIC 3168, Australia

**Keywords:** HSF1, epitope mapping, ovarian cancer peptides, IgA, biomarkers

## Abstract

**Simple Summary:**

Most women are diagnosed with high-grade serous ovarian cancer (HGSOC) at stage III, when the cancer has already spread, contributing to poor survival outcomes. However, while earlier diagnosis increases survival rates, there is a lack of early diagnosis biomarkers. Previously, autoantibodies specific for phosphorylated heat shock factor 1 (HSF1-PO4) were suggested as a potential diagnostic biomarker for early-stage HGSOC. In the present study, specific regions within HSF1 were identified, tested and confirmed as useful biomarkers, with comparable diagnostic potential to the full protein, across two separate clinical cohorts. Additionally, antibody responses to HSF1-PO4 and the corresponding peptides were found to increase following a round of standard first-line chemotherapy. Together, our data suggest that the identified short peptide sequences could be used as practical alternatives to support early diagnosis or monitor responses to chemotherapy.

**Abstract:**

Autoantibodies recognising phosphorylated heat shock factor 1 (HSF1-PO4) protein are suggested as potential new diagnostic biomarkers for early-stage high-grade serous ovarian cancer (HGSOC). We predicted in silico B-cell epitopes in human and murine HSF1. Three epitope regions were synthesised as peptides. Circulating immunoglobulin A (cIgA) against the predicted peptide epitopes or HSF1-PO4 was measured using ELISA, across two small human clinical trials of HGSOC patients at diagnosis. To determine whether chemotherapy would promote changes in reactivity to either HSF1-PO4 or the HSF-1 peptide epitopes, IgA responses were further assessed in a sample of patients after a full cycle of chemotherapy. Anti-HSF1-PO4 responses correlated with antibody responses to the three selected epitope regions, regardless of phosphorylation, with substantial cross-recognition of the corresponding human and murine peptide epitope variants. Assessing reactivity to individual peptide epitopes, compared to HSF1-PO4, improved assay sensitivity. IgA responses to HSF1-PO4 further increased significantly post treatment, indicating that HSF1-PO4 is a target for immunity in response to chemotherapy. Although performed in a small cohort, these results offer potential insights into the interplay between autoimmunity and ovarian cancer and offer new peptide biomarkers for early-stage HGSOC diagnosis, to monitor responses to chemotherapy, and widely for pre-clinical HGSOC research.

## 1. Introduction

Heat shock factor 1 (HSF1) is the key regulator of the heat shock response, where it drives transcription of many target genes. Highly conserved across eukaryotic species, sharing approximately 84% sequence identity between human, mouse and bovine [1], HSF1 has been implicated in cell differentiation [2], development [3], and aging [4]. Moreover, HSF1 is associated with neurodegenerative diseases and cancers [5,6,7,8,9,10]. Defects in HSF1 exacerbate protein misfolding and aggregation in neurodegeneration diseases such as Parkinson’s disease or Alzheimer’s dementia [5,6]. In contrast, it is suggested that HSF1 supports and contributes to tumour growth, proliferation and metastasis [7,8,9,10]. In clinical studies, increased HSF1 expression levels are correlated with poor prognosis and advanced metastasis in breast cancer [11,12], non-small cell lung cancer [13], gastric cancer [14], oesophageal squamous cell cancer [15], ovarian cancer [16] and others. High expression of HSF1 has additionally been associated with cancer stem cells [16], as well as drug resistance [12]. For these reasons, HSF1 has been proposed as a potential target for therapies.

High-grade serous ovarian cancer (HGSOC) has the highest mortality rate of any of the gynaecological cancers. Not only are women diagnosed late (with most women diagnosed at stage III) [17], prognosis remains poor, with the five-year survival rate less than 50% [18]. Currently, blood serum levels of cancer antigen-125 (CA-125) are used to assist in diagnosis of HGSOC as well as to monitor recurrence following treatment [19]. However, CA-125 is not specific to ovarian cancer and may be found at normal levels in ~50% of women in early stage of disease [20,21,22]. For this reason, new tools are required for both diagnostic and prognostic monitoring.

Circulating autoantibodies (AAb) targeting tumour-associated antigens (TAAs) have been explored as potential biomarkers for cancer diagnosis [23,24,25,26]. The presence of AAb has been well documented in multiple cancer types [23,24,25,26]. However, these are typically of the IgG isotype [23,24,25,26]. In a prior pilot study [27], we performed large-scale screening of IgM, IgG and IgA AAbs in early- and late-stage HGSOC patients and identified an increase in IgA-specific AAbs to phosphorylated HSF1 (HSF1-PO4) in HGSOC patients, with a significant increase in the early stages, in comparison to control cohorts [27].

While immune responses to heat shock proteins (the downstream targets of HSF1) have been reported [28,29,30,31], much less is known about immune responses to HSF1. In this study, we evaluated new, potentially useful B-cell epitopes present in HSF1. IgA-specific AAb responses to HSF1-PO4 were tested in HGSOC patients (stages I, III and IV), benign serous cystadenoma patients, and healthy controls. Due to the conserved nature of HSF1 [1], these responses were compared to responses towards predicted B-cell epitopes corresponding to both human and murine sequences. Herein, we identify multiple novel B-cell epitopes to help assess antibody responses against HSF1-PO4, with the potential to be used across both human studies and in animal models.

## 2. Materials and Methods

### 2.1. Patient Samples

Two independent clinical cohorts were utilised for these studies. Cohort 1 (Table 1) comprised the same patient samples analysed in our previous study identifying AAb against HSF1-PO4 as a potential biomarker, and all details are as previously described [27]. Briefly, EDTA-chelated plasma samples were accessed from biobanked samples. Samples were collected from women undergoing surgery for suspected gynaecological malignancy between 2007 and 2012. This study was approved by the Monash Health Human Research Ethics Committee (HREC certificates #06032C, #02031B) and all participants provided prior informed written consent.

Testing was then expanded to a larger cohort (Cohort 2, Table 2), comprising clinical samples collected between 2014 and 2017 as a part of Project 13/32, HREC of Royal Women’s Hospital, Melbourne, Australia [32]. Sera and plasma were obtained from venous blood samples collected at diagnosis, prior to surgery and prior to cycles of chemotherapy (pre-cycle). Patients also treated with Bevacizumab during first-line treatment continued a Bevacizumab-only treatment for an additional 6 months following the sixth cycle of primary treatment (pre-cycle 7–12). All relevant clinical information including demographic status, medical and drug history, clinical diagnosis, stage and disease extent, status and follow-up data were collected. Venous blood samples were also collected from women diagnosed with benign serous cystadenoma, as well as women with no pathology but who were undergoing risk reduction surgery for a known genetic mutation or a strong family history of ovarian and/or breast cancers.

### 2.2. Peptide Design and Synthesis

**Figure 1:**
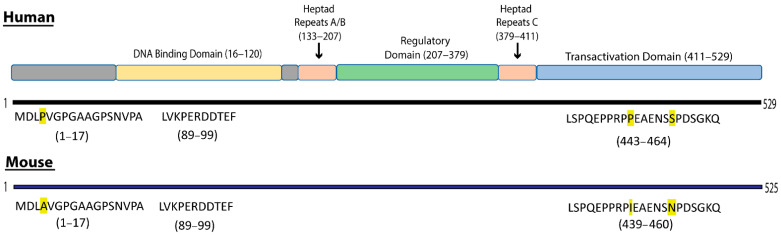
Human HSF1 (HuHSF1) structure contains four main domains: the DNA-binding domain, heptad repeats, the regulatory domain and the transactivation domain [33]. HuHSF1 contains 529 amino acids (aa) in the sequence and mouse HSF1 (MuHSF1) contains 525aa. Three locations in the human sequence were selected for further testing: aa1–17, aa89–99 and aa443–464. The orthologous sequences in MuHSF1 were also selected (aa1–17 and aa439–460). Highlighted residues represent the change of residue between human and mouse sequences. Human and mouse sequence variants have a 94.1, 100, and 90.1% similarity for aa1–17, aa89–99 and aa443–464, respectively.

Whole protein sequence of HSF1 for both *Homo sapiens* (Q00613) and *Mus musculus* (P38532) was obtained from UniProt Knowledgebase database (https://www.uniprot.org, accessed on 3 April 2019). B-cell epitope prediction was performed in silico using the Immune Epitope Database and Analysis Resource antibody epitope prediction tool (IEDB, http://tools.iedb.org/bcell/, accessed on 3 April 2019). Orthologous regions between *Homo sapiens* and *Mus musculus* were identified using the National Center for Biotechnology Information (NCBI) standard protein blast (https://blast.ncbi.nlm.nih.gov/, accessed on 8 April 2019). Epitope sequences of interest (Figure 1) were synthesised at a 95% purity by Mimotopes Pty Ltd. (Clayton, Australia).

### 2.3. Enzyme-Linked Immunosorbent (ELISA) Assay

Phosphorylated and non-phosphorylated synthetic peptides or recombinant HSF1-PO4 protein (Abcam #115508) were diluted in carbonate/bicarbonate coating buffer (4 µg/mL) and incubated overnight in 96-well NUNC maxisorp plates at 4 °C. Plates were washed with wash buffer (PBS/0.05% *v*/*v* Tween-20) and blocked with PBS/1% BSA for a minimum of 1 h at 37 °C. After washing, plasma/sera were added (duplicate wells) and incubated at 4 °C overnight (1:10 or 1:20 dilution in wash buffer). After washing, HRP-conjugated goat anti-human IgA alpha chain (1:10,000, Abcam #98558) was added and incubated at 37 °C for 1.5 h. Following washing, the reaction was developed using TMB substrate (Invitrogen, Waltham, MA, USA) and stopped with 1 M HCl. Absorbance was immediately read at 450 nm (Optical Density (OD)_450nm_) using a Multiskan Go plate reader (Thermo Scientific, Waltham, MA, USA). Where specified, OD_450nm_ values were normalised against values from a single patient sample consistently run across multiple experiments to account for inter-assay variability. The selected sample (OV0023, refer to Supplementary Table S2) had a mid-range anti-HSF1-PO4 or anti-peptide response (ranging OD_450nm_ 0.41–1.0, depending on the target) and was thus chosen to avoid extreme measurements that could introduce bias.

### 2.4. Statistical Analysis

Statistical significance was assessed using GraphPad Prism v8.0.0. Normality was tested by the Anderson–Darling test prior to assessing significance using the Mann–Whitney U test or a paired *t*-test as appropriate, for non-normal distributed and normal distributed data, respectively. For correlation analysis, data were log transformed to establish normality, and Pearson’s correlation analysis was performed. Significance is shown as *p* < 0.05 (*) or *p* < 0.01 (**) and data are shown as either the median and IQR or the mean ± SEM, as indicated in figure legends. All group sizes and specific statistical analyses are additionally indicated in the figure legends. 

## 3. Results

### 3.1. Definition of Selected B-Cell Epitopes in Heat Shock Factor 1

To study the immune response towards HSF1, we first used IEDB to predict potential B-cell epitopes within the HSF1 sequence. Peptide sequences with a minimum prediction score of 1.5 and a maximum of 3.5 were selected for further analysis. Sequence 1–17 (score 2) was selected for further study as the majority of this sequence lies outside of the four key domains outlined (the DNA-binding domain, heptad repeats, the regulatory domain and the transactivation domain, Figure 1) in the human sequence [33,34,35]. Sequence 89–99 (score 1.5) is identical in human and mouse sequences and sits within the DNA-binding domain, a region less explored as having target immunogens. The final sequence 443–464 (score 3) is in a region of the protein targeted by some commercial antibodies specific for HSF1. As HSF1 is highly conserved [1], to explore whether cross-reactivity occurs for potential utility in ovarian cancer animal models, the orthologous murine sequences were identified using the NCBI protein blast, and the corresponding peptides also synthesised (Figure 1).

### 3.2. Designed Synthetic Peptides Have a Positive Correlation to HSF1-PO4 Antibody Responses

**Figure 2:**
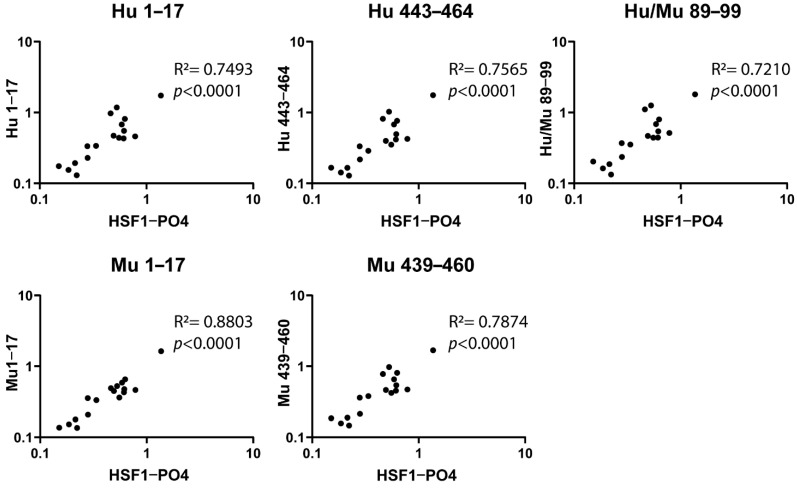
IgA-specific antibodies to HSF1-PO4 and the individual selected epitopes, were measured by indirect ELISA. Plasma samples (diluted 1:20) from control samples (*n* = 10) and early-stage HGSOC patients (*n* = 7), were analysed in duplicate. Associations between responses were analysed using Pearson’s R correlation (R^2^), following log transformation to establish normality (data found in Supplementary Table S1). Individual OD values plotted on a log scale.

Previously, IgA-specific AAbs targeting HSF1-PO4 were found to be significantly elevated in a small cohort of early-stage HGSOC patients, in comparison to controls [27]. Using samples from this study (cohort 1), which have a range of IgA-specific anti-HSF1-PO4 responses, we compared the IgA-specific antibody responses of the synthesised non-phosphorylated peptides to those of full-length HSF1-PO4 protein, using indirect ELISA. While no differences were detected between controls and early-stage HGSOC (Supplementary Figure S1), a significant positive correlation was observed between responses to all peptides examined and to HSF1-PO4 (Figure 2) suggesting that the selected epitopes are an appropriate surrogate for full-length HSF1-PO4 to assess the IgA-specific AAb response against HSF1-PO4.

### 3.3. Phosphorylation of Peptides Does Not Affect the Antibody Responses to the Predicted Epitopes

In our pilot study that identified increased anti-HSF1 AAbs in early-stage HGSOC patients [27], the increased response was specifically to the phosphorylated full-length protein and not to the non-phosphorylated protein. However, the same patient samples used in this study showed significant positively correlated responses between non-phosphorylated peptides and the full-length phosphorylated protein (Figure 2). Therefore, to identify whether phosphorylation of the epitopes may impact the antibody responses, the human-specific epitopes were synthesised with phosphorylated serine and threonine residues. Sequence Hu 1–17 and Hu/Mu 89–99 contain one phosphorylation site, whereas sequence Hu 443–464, containing three serines, was phosphorylated at each site. 

**Figure 3:**
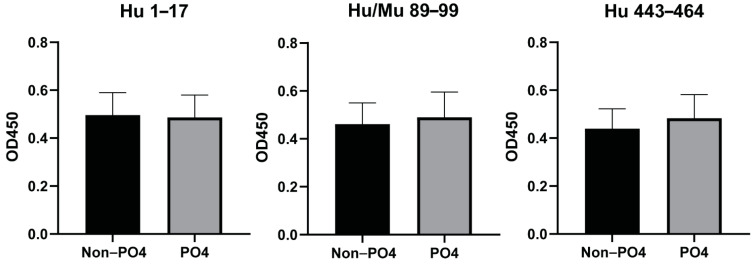
IgA-specific antibodies to individual phosphorylated (PO4) or non-phosphorylated (Non-PO4) epitopes, via indirect ELISA. Using a subset of cohort 2 (*n* = 8), plasma samples (diluted 1:20), collected at varied timepoints (*n* = 9) were analysed in duplicate. Timepoints include pre-surgery, pre-cycle 2, 5, 8, 10, 11, 12 and following surgery that occurred during chemotherapy treatment. Data are represented as the mean ± SEM and significance was tested using a paired *t*-test.

With the majority of women diagnosed at stage III [17], we further wanted to explore the anti-HSF1 AAb responses in a larger cohort of advanced-stage HGSOC patients, and corresponding benign serous cystadenoma and healthy controls (cohort 2). A subset of these HGSOC patients (*n* = 8) were used to identify any effect of phosphorylated residues within the predicted epitopes. Plasma samples collected at varied timepoints during chemotherapy treatment (pre-surgery, pre-cycle 2, 5, 8, 10, 11, 12) were used to measure and compare IgA-specific antibody responses between phosphorylated and non-phosphorylated human epitopes (Figure 3). For each of the epitopes (Hu 1–17, Hu/Mu 89–99 and Hu 443–464), there were no significant differences between the antibody responses to between the phosphorylated and non-phosphorylated epitopes. Due to this, the non-phosphorylated peptides were used in all consecutive experiments. 

### 3.4. Correlation Validation between Responses to HSF1-PO4 and Predicted Peptide Epitope and the Assessment of Species Cross-Reactivity

**Figure 4:**
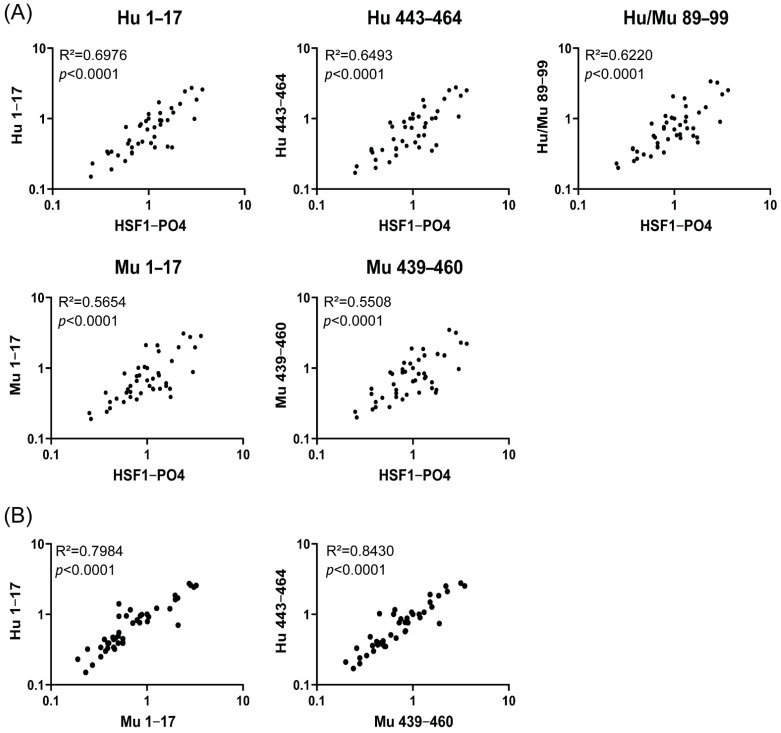
IgA antibody responses to anti-HSF1-PO4 and predicted epitopes in cohort 2 patients (*n* = 45). Responses in sera (diluted 1:10) from patients with no pathology (*n* = 11), benign serous cystadenoma (*n* = 9) and late stage HGSOC (total *n* = 25, diagnosis/pre-surgery = 16, pre-cycle 1 = 6, pre-cycle 2 = 3) were measured via indirect ELISA and normalised to a mid-responder (OV0023). OD_450nm_ for mid-range responder as follows: HSF1-PO4 (0.41 or 0.69); Hu 1–17 (0.95); Hu 443–464 (1.0); Hu/Mu 89–99 (0.54 or 0.58); Mu 1–17 (0.52 or 0.62); Mu 439–460 (0.51 or 0.68). (**A**) Pearson’s R correlation (R^2^) and *p*-value of normalised peptide responses to HSF1-PO4 responses plotted on a log scale. (**B**) R^2^ and *p*-value of normalised responses of corresponding human and mouse sequences, plotted on a log scale. R^2^ shown for Mu 1–17, although dataset did not pass normality test (Supplementary Table S2).

To further explore the utility of the peptides in comparison to the protein, we next evaluated anti-HSF1-PO4 AAb responses in cohort 2. Sera collected from the control groups (benign and healthy) and from mixed timepoints (Diagnosis/Pre-Surgery, Pre-Cycle 1 and Pre-Cycle 2) of malignant patients were used. Normalised IgA-specific AAb responses to HSF1-PO4 were compared to normalised peptide responses (Figure 4A). As observed in cohort 1, significant positive correlations were found between Ab responses to all the synthetic peptides examined and HSF1-PO4. 

In both cohorts of patients, we observed strong correlations between the responses to mouse-specific epitopes and responses against human HSF1-PO4. As HSF1 is highly conserved between species [1], and the orthologous sequences have one or two amino acid differences, we compared the AAb responses in the mouse and human orthologous sequences (Figure 4B). In both sequence locations strong, positive correlations were observed: Hu443 vs. Mu439 (r = 0.9181, R^2^ = 0.8430, *p* < 0.0001) and aa1–17 (r = 0.8935, R^2^ = 0.7984, *p* < 0.0001) (Figure 4B). These findings indicate the occurrence of cross-reactive antibody responses. 

### 3.5. Sensitivity Assays Show Predicted Epitopes with Equal or Higher Measurements in Comparison to HSF1-PO4

**Figure 5:**
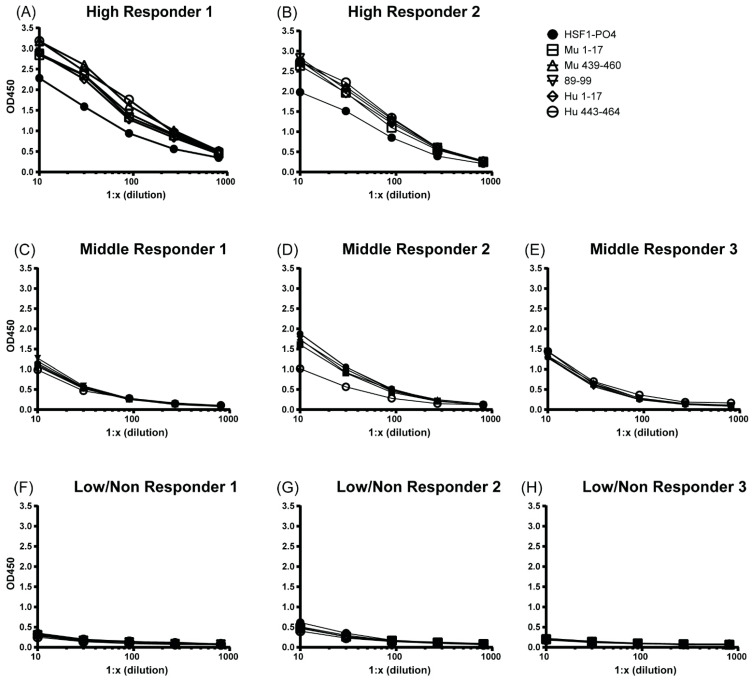
Sensitivity of IgA antibodies to HSF1-PO4 and individual epitopes, at multiple sample dilutions, measured via indirect ELISA. Three-fold serial dilution, of serum, from 1:10 (1:30, 1:90, 1:270 and 1:810), run in duplicate, of individual samples from two high (**A**,**B**), three mid (**C**–**E**) and three low (**G**–**H**) responders to HSF1-PO4. Key is representative of all figures (**A**–**H**). All open symbols represent peptides and filled in circle represents HSF1-PO4.

To further compare the IgA-specific antibody responses of the peptides to that of HSF1-PO4, we explored sensitivity of the responses by titrating samples from individuals with a high, medium or low/no response towards full HSF1-PO4 (total 8 individuals from cohort 2), and measured IgA-specific responses (Figure 5).

In the two high responding individuals to HSF1-PO4, responses to all five peptide sequences, whether derived from the human or mouse HSF1 sequence, were higher than the responses to human HSF1-PO4 (Figure 5A,B). In High Responder 1 (Figure 5A), the OD_450nm_ reading remained clearly higher at dilutions 1:10–1:270. Whereas High Responder 2 had higher responses to the peptides at the lower dilutions (1:10, 1:30 and 1:90) but were not as sensitive at the highest dilutions (1:270 and 1:800).

Two of the three mid-level responders had similar responses at all sera dilutions, for HSF1-PO4 and all peptides (Figure 5D–F). The remaining mid-responder (Mid responder 2, Figure 5D) had visually higher responses in the peptides in comparison to HSF1-PO4 at the lower dilutions (1:10 and 1:30). As the dilutions increased, the difference of OD readings between the protein and peptides decreased and became similar. All low/non-responders, whether measuring full length HSF1 or peptides, remained low (Figure 5G–I). These results (as well as area under the curve analysis (Supplementary Table S3)) further suggest that any of the described peptides may be used in the place of full length HSF1 protein to measure antibody responses. 

### 3.6. Patients with Advanced HGSOC Have Decreased Anti HSF1-PO4-Specific AAb Responses

**Figure 6:**
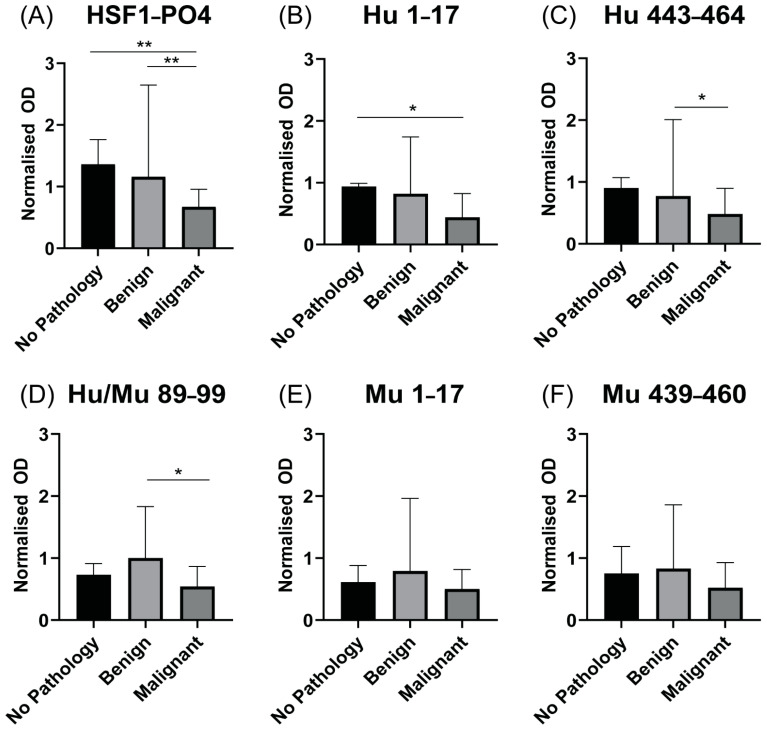
IgA-specific antibodies against (**A**) HSF1-PO4 and (**B**–**F**) predicted epitopes measured via indirect ELISA. Serum samples (diluted 1:10) from Cohort 2 patients (no pathology *n* = 11, benign *n* = 9 and malignant *n* = 23), where malignant samples were collected prior to chemotherapy treatment, were ran in duplicate. All readings were normalised to a mid-range responder consistently run across multiple experiments. OD_450nm_ for mid-range responder: HSF1-PO4 (0.41 or 0.69); Hu 1–17 (0.95); Hu 443–464 (1.0); Hu/Mu 89–99 (0.54 or 0.58); Mu 1–17 (0.52 or 0.62); Mu 439–460 (0.51 or 0.68). As normality tests showed non-normal distributions, data are presented as the median and IQR and Mann–Whitney U statistical analysis was performed, * *p* < 0.05 and ** *p* < 0.01.

As most women with HGSOC are diagnosed at the late stage of disease [17], IgA-specific AAb responses against HSF1-PO4 and synthetic peptides were assessed in cohort 2 as potential diagnostic biomarkers. Unexpectedly, we observed a significant (*p* < 0.01) reduction in anti-HSF1-PO4 reactivity in patients with advanced HGSOC relative to both benign serous cystadenoma and healthy controls (Figure 6A). The pattern of responses against HSF1-PO4 observed, in which AAb readings were highest in no pathology samples, followed by the benign cystadenoma group and finally late stage malignant patients, were consistent in the human only peptides (Figure 6B,C). Although this same trend was followed, the two peptides Hu 1–17 and Hu 443–453 only showed a significant difference (*p* = 0.0366 and 0.0305, respectively) to either the no pathology or the benign cystadenoma group, respectively. Responses to peptides Hu/Mu 89–99, Mu 1–17 and Mu 439–460 (Figure 6D–F) showed a different pattern of response where AAb readings were highest in benign cystadenoma samples, followed by the no pathology group and then malignant samples. Of these, only Hu/Mu 89–99 showed a significant difference (*p* = 0.0410) between the malignant group and the benign serous cystadenoma group (Figure 6D). 

### 3.7. Increased IgA-Specific AAb Responses to HSF1 following a Cycle of Chemotherapy

**Figure 7:**
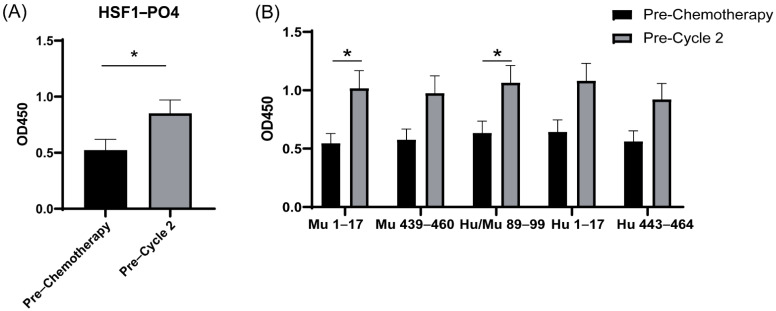
IgA-specific antibody responses in patients at diagnosis and prior to cycle 2 of first-line chemotherapy (*n* = 10). Sera (diluted 1:10) IgA responses against (**A**) HSF1-PO4 or (**B**) predicted epitopes. Data are shown as the mean ± SEM from duplicates. A paired T-test was performed, * *p* < 0.05.

Upon diagnosis, women typically undergo a cytoreductive surgery followed by six cycles of combination chemotherapy consisting of carboplatin and paclitaxel [36]. Due to cytotoxic effects and thus stress-induced environments resulting from chemotherapies, which have been shown in other cancers to increase HSF1 [12] and heat shock proteins [37], we further explored the effect of chemotherapy on antibodies targeting HSF1. We therefore assessed the influence of chemotherapy on AAb in a group of patients (*n* = 10), prior to and then following a single cycle of chemotherapy (Pre-Cycle 2). Following one cycle of chemotherapy, a significant increase in antibodies targeting HSF1-PO4 was observed (*p* = 0.047) (Figure 7A), identifying HSF1-PO4 as a tumour-associated antigen following chemotherapy. 

To further evaluate the ability of the peptides to measure the influence chemotherapy has on the IgA-specific antibody response to HSF1, AAb levels towards the peptides were assessed (Figure 7B). There was an increase in antibody responses to all sequences following one cycle of chemotherapy. Of these, Mu1–17 and Hu/Mu89–99 showed significant increases (*p* = 0.029 and *p* = 0.048, respectively). This reinforces previous findings of cross-reactive immune responses occurring as well as the potential to use these peptides in place of full-length HSF1-PO4 to measure AAb responses to track changes due to cycles of chemotherapy.

## 4. Discussion

AAbs are an appealing biomarker for both cancer diagnosis and prognosis as they can be easily measured from minimally invasive blood collection, and have shown increased levels in the early stages of cancer (reviewed in [38]). As AAbs to HSF1-PO4 were previously identified as a potential early-stage diagnostic biomarker in HGSOC [27], the present study sought to identify and investigate novel B-cell epitopes in HSF1. Five non-phosphorylated peptides of human and mouse origin were tested, and IgA responses to these epitopes were found to be significantly positively correlated with reactivity to HSF1-PO4, in two separate clinical cohorts. Further testing found no differences of responses between phosphorylated and non-phosphorylated peptides. Additionally, in mid-high responders, the IgA AAb responses were higher towards the peptides than the full-length protein sequence. These findings show that the peptides representing predicted B-cell epitopes may be used as surrogates to measure the anti-HSF1-PO4 antibody responses. Finally, for the first time, we show increased anti-HSF1-PO4 IgA antibody responses in a small cohort of advanced HGSOC patients following a single round of standard first-line chemotherapy.

Here, we explored three locations of the human HSF1 sequence for the chosen peptides. Selected due to its high prediction score, sequence 443–464 targets the transactivation domain of the protein, where several commercially available anti-HSF1 antibodies are available that target as the immunogen. However, the exact epitope recognised by these antibodies is not provided by the manufacturers. Other commercial antibodies only target sequences in the regulatory domain and heptad repeats-C region. Therefore, we chose to explore the existence of epitopes that may exist outside these regions. Sequence 89–99 was found to be the only predicted epitope within the DNA-binding domain. Furthermore, its 100% homology to the corresponding mouse sequence allows for translation into animal models for future studies. Finally, sequence 1–17 was within a highly conserved region, outside any of the previously defined functional domains [33,34,35]. Despite these different locations, antibody responses to all tested peptides significantly correlated to the HSF1-PO4 responses. No single peptide epitope was substantially more correlated to HSF1 than any other, suggesting that any of the sequences could be used as a surrogate to measure the anti-HSF1-PO4 responses. This was further shown in the sensitivity assays, where all curves followed a similar pattern irrespective of the antigen. Within a sample of cohort 2, our data show that the antibodies to HSF1-PO4 and all peptides bound with similar affinity, no matter whether initial responses were low, middle or high range. We additionally observed no differences between antibody responses to our phosphorylated and non-phosphorylated peptides at timepoints throughout chemotherapy, suggesting that phosphorylation does not affect antibody recognition. This, however, contradicts previous findings which found that the increased responses were to the recombinant HSF1-PO4 protein and not to non-phosphorylated HSF1. Given the limitations imposed by sample availability in this study, it would be beneficial in the future to perform a comparison study between non-phosphorylated and phosphorylated protein and peptides at all timepoints, and explore the antibody affinities, for all patients.

CA-125, the current biomarker used for HGSOC diagnosis, is not specific for ovarian cancer [20,21,22]. For this reason, several studies identifying AAbs in ovarian cancer screen patients at diagnosis to compare or combine levels of AAb with CA-125 to increase diagnostic capabilities [24,27,39,40]. Given antibody responses to HSF1-PO4 and peptides correlated in the same cohort which previously identified the potential diagnostic value of anti-HSF1-PO4 in early-stage HGSOC patients [27], these peptides have the potential to be used for future studies further investigating the diagnostic value of anti-HSF1-PO4 within a larger cohort. In advanced-stage HGSOC, where most women are diagnosed [17], we identified levels of IgA-specific AAb targeting HSF1-PO4 were decreased, in comparison to the control groups. Indicating that IgA-specific AAb to neither HSF1-PO4 nor the synthetic peptides are effective as diagnostic markers at late-stage disease. However, IgA levels to HSF1-PO4 increased after a cycle of combined platinum- and taxol-based chemotherapy (carboplatin and paclitaxel), identifying HSF1-PO4 as a potential tumour-associated antigen. Alternative explanations for the increase in anti-HSF1-PO4 antibodies may be due to a reaction to chemotherapy and an increase in antigen. HSF1 expression has been shown to increase following treatment with carboplatin [12]. This high expression was found to contribute to the protection of tumour cells from death following chemotherapy by its role in autophagy, a cellular process allowing cells to survive stressful conditions [12]. While a statistically significant increase was observed post a cycle of chemotherapy, the findings are limited by the small sample size, and it would be valuable to confirm these trends in additional separate studies and to further explore the utility of antibody responses to both full HSF1-PO4 and peptides. While more than 80% of HGSOC patients respond to initial treatment, recurrence is just as common (>80%) [41] and the duration generally varies with time [42,43]. Patients who recur within 6 months of first-line treatment are considered resistant and have a poor survival prognosis, while tumours that recur after 6 months are platinum sensitive and these patients are considered to have a good prognosis [44]. Following further studies in a larger cohort of patients, each of the identified peptide sequences may be able to provide a practical method to monitor the chemotherapy response. Future larger studies looking at the role of these peptides in discriminating patients that respond or do not respond to first-line treatment (in Australia being surgery followed by combination carboplatin/paclitaxel chemotherapy) may help to individualise therapy for patients with this lethal disease. In the present pilot study, we did not have the statistical power to compare the antibody responses to HSF1-PO4 and peptides in the low-, middle- and high-range responders with chemotherapy response. Future studies utilising these peptides may look at correlating responses to patient outcome and tumour burden. This could be performed through monitoring the antibody responses at later cycles of chemotherapy and comparing these to patient disease and survival outcome. These may allow for prognostic markers that indicate whether a patient may require increased monitoring, or a modification of their treatment regimen.

Due to the highly conserved nature of HSF1 [1], the orthologous peptide sequences from the mouse HSF1 sequence were additionally synthesised and tested for IgA-specific antibody responses in the human cohorts. As with the human peptide sequences, we observed significant positive correlations to the HSF1-PO4 responses. These strong correlations were also observed when comparing the responses of the murine to the human-specific epitopes. These findings indicate that cross-reactive antibody responses occur, which was further validated by the significantly increased antibody responses to Mu 1–17 and Hu/Mu 89–99 following a single cycle of chemotherapy. With this presence of cross-reactivity, epitopes Mu 1–17 and Hu/Mu 89–99, given the responses in a small cohort, could be used with an ovarian cancer mouse model to further explore the role of the anti-HSF1-PO4 immune response. Future studies in an ovarian cancer mouse model may be used to monitor the anti-HSF1-PO4 responses to different therapies for prognostic outcomes, particularly in cases where access to clinical samples may be limited.

Our focus looking at IgA-specific responses to HSF1 was due to the increase in IgA to HSF1 in HGSOC following the screening of IgA, IgM and IgG [27]. A limitation of this pilot study was its limited size, and additionally, when using a larger cohort, it may also be informative in future studies to expand the study to other Ig isotypes, for example IgG, as a measure of secondary peripheral responses. Interestingly, a recent study has highlighted the protective role of IgA antibodies in HGSOC [45]. In it, the authors suggest that augmenting B-cell responses may be more effective in the anti-tumour response. As HSF1 has been implicated in several cancer types [11,12,13,14,15,16], it has been proposed as a potential anti-cancer therapy target [46,47]. Targeting HSF1 as a therapy in cancers is indeed a growing field. Silencing HSF1 in melanoma cancer cells showed a reduction in cell proliferation through cell cycle arrest and an increase in apoptosis [10]. Similarly, a nucleotide analog which exhibited anti-cancer effects has been shown to downregulate HSF1 in both pancreatic and ovarian cancer studies [48,49]. The increased antibody responses to the predicted peptides in the present study identify these sequences as targets of the immune system. Using animal models, the peptides may be further explored as potential vaccine or antibody therapeutic targets, alone or in combination with chemotherapy, to boost the anti-tumour immune response and potentially provide a beneficial outcome.

## 5. Conclusions

In the present study, two separate HGSOC cohorts consistently showed that IgA responses to five peptide sequences, mapped within HSF1, correlate with responses against HSF1-PO4 full protein. To enable clinical translation and further explore the role for diagnostic purposes in early stages of disease, or monitor changes due to chemotherapy, a large-scale study would be required. Overall, these findings show that each of the potential peptides may be used in place of the full-length HSF1-PO4 protein to measure antibody responses. Proteins can often be less accessible as a diagnostic/prognostic reagent due to the relatively high costs required to produce them with sufficient purity. Therefore, these identified peptide sequences, particularly the non-phosphorylated versions, may provide increased practicality and cost effectiveness to further explore anti-HSF1-PO4 immune responses. These may be used as therapeutics and as biomarker targets to monitor prognostic changes due to treatment, in both human and animal models of HGSOC.

## Figures and Tables

**Table 1:** Summary of Cohort #1.

Group/Disease Status	Stage of Disease	#Patient	Median Age (Range)
Healthy Controls	n/a	*n* = 10	59.5 (56–65.5)
Early Stage	I (a–c)	*n* = 8	63.0 (53–86)

**Table 2:** Summary of Cohort #2.

Group/Disease Status	Stage of Disease	#Patient	Median Age (Range)
Malignant	III (*n* = 23), IV (*n* = 2)	*n* = 25	60 (49–83)
Benign serous cystadenoma	n/a	*n* = 9	52 (35–70)
No pathology	n/a	*n* = 11	45 (42–59)

## Data Availability

The data presented in this study are available within the article/supplementary material.

## Supplementary Materials

The following are available online at https://www.mdpi.com/article/10.3390/cancers13164201/s1, Figure S1: Cohort 1 IgA specific antibodies to HSF1-PO4 and the individual selected epitopes; Table S1: Raw OD and log-transformed data and results of normality tests in cohort 1; Table S2: Raw OD and log-transformed data and results of normality tests in cohort 2; Table S3: Area under the curve (AUC) analysis of sensitivity between HSF1-PO4 and peptides.
