# Supplementary material for: Mapping Epitopes Recognised by Autoantibodies Shows Potential for the Diagnosis of High-Grade Serous Ovarian Cancer and Monitoring Response to Therapy for This Malignancy
Source: Cancers (Basel). 2021 Aug 20;13(16):4201. doi: 10.3390/cancers13164201 (PMC8392293; doi:10.3390/cancers13164201)
Supplement: Supplementary file 1 [file cancers-13-04201-s001.zip › cancers-1261399_sup-proof-updated.pdf]

cancers

MDPI

Article

Mapping Epitopes Recognised by Autoantibodies Shows Potential for the Diagnosis of High-Grade Serous Ovarian Cancer and Monitoring Response to Therapy for This Malignancy

Rhiane Moody [1], Kirsty Wilson [1], Nirmala Chandralega Kampan [2], Orla M. McNally [3], Thomas W. Jobling [4], Anthony Jaworowski [1], Andrew N. Stephens [5,6] and Magdalena Plebanski [1,*]

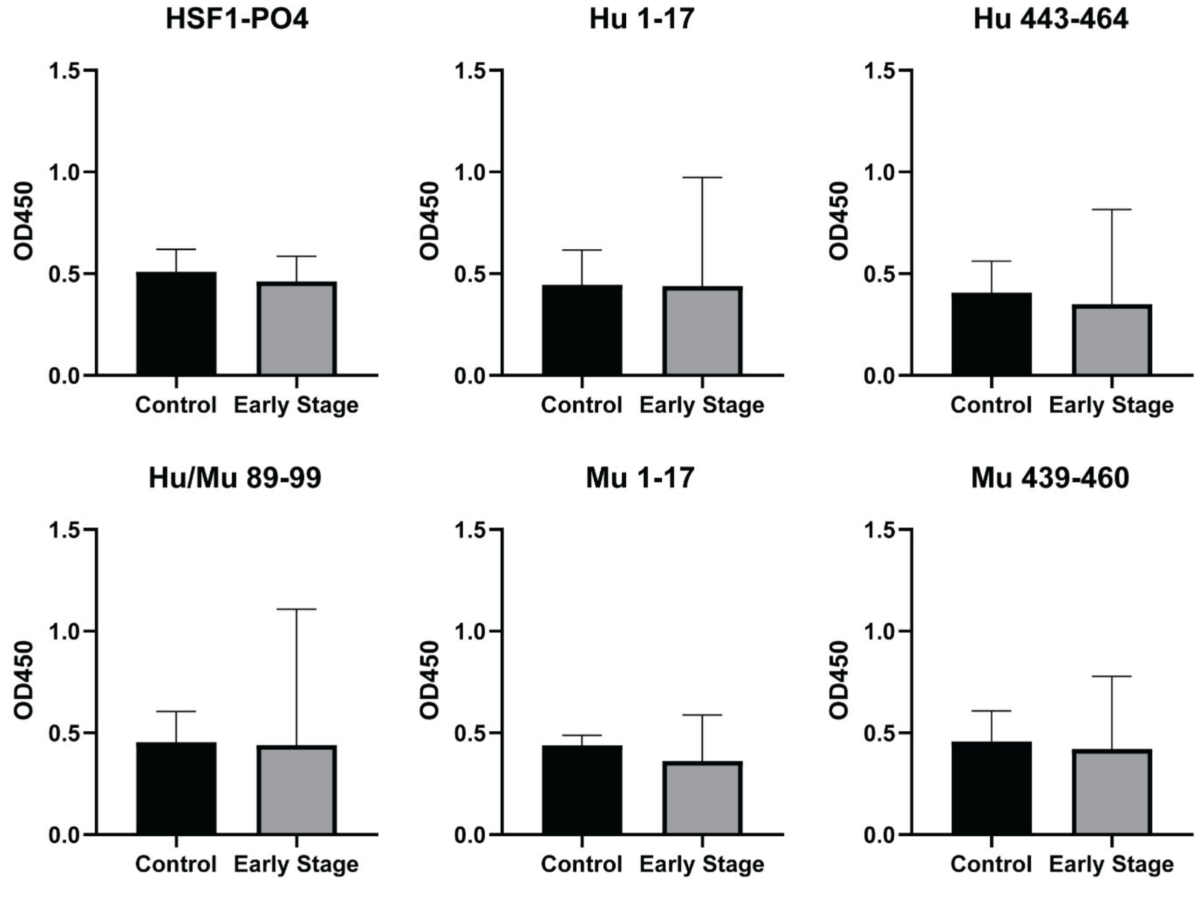

Figure S1. Cohort 1 IgA specific antibodies to HSF1-PO4 and the individual selected epitopes, were measured by indirect ELISA. Plasma samples (diluted 1:20) from control samples (n=10) and early stage HGSOC patients (n=7), were analysed in duplicate. Data presented as median with IQR and significance tested using Mann-Whitney U test.
